# Nutritional and 13-Week Subchronic Toxicological Evaluation of *Lignosus rhinocerotis* Mycelium in Sprague-Dawley Rats

**DOI:** 10.3390/ijerph18031271

**Published:** 2021-01-31

**Authors:** I-Chen Li, Bi-Hua Yang, Jing-Yi Lin, Shan Lin, Chin-Chu Chen

**Affiliations:** 1 Biotech Research Institute, Grape King Bio Ltd., Taoyuan City 325, Taiwan; ichen.li@grapeking.com.tw (I.-C.L.); sybil.yang@grapeking.com.tw (B.-H.Y.); jingyi.lin@grapeking.com.tw (J.-Y.L.); shan.lin@grapeking.com.tw (S.L.); 2 Department of Food Science, Nutrition, and Nutraceutical Biotechnology, Shih Chien University, Taipei City 104, Taiwan; 3 Institute of Food Science and Technology, National Taiwan University, Taipei City 106, Taiwan; 4 Department of Bioscience Technology, Chung Yuan Christian University, Taoyuan City 320, Taiwan

**Keywords:** *Lignosus rhinocerotis*, mycelia, umami index, 13 week toxicity, NOAEL

## Abstract

*Lignosus rhinocerotis* (Tiger’s Milk mushroom) is a novel mushroom with sclerotium belonging to the *Polyporaceae* family and has been reported widely to possess anti-cancer, anti-cough, antioxidant, gastro-protective, immuno-modulating, and neurite-stimulating properties. As numerous studies have proven the tremendous medicinal values of *L. rhinocerotis*, it is necessary to understand its nutrition as well as its safety for the recipient. Previous research on *L. rhinocerotis* has mainly focused on the naturally occurring sclerotium and may have overlooked mushroom mycelia from submerged liquid fermentation, which ensures a high uniform quantitative biomass production as well as a high biological value. Hence, this is the first report on the evaluation of nutrition and 13-week repeated oral toxicity of *L. rhinocerotis* mycelium (LRM). The LRM powder contained 9.0 ± 4.2% moisture, 1.9 ± 1.3% ash, 1.6 ± 2.2% crude lipid, 8.4 ± 5.3% crude protein, 79.3 ± 4.6% carbohydrate, and 364 kcal/100 g energy. The total free amino acid ranged from 349 to 5636 mg/100 g and the umami index of freeze-dried LRM powder was 0.37. For safety assessment, ninety-six rats were divided into four groups, each consisting of twelve male and twelve female rats. Test articles were administered by oral gavage to rats at 850, 1700, and 3400 mg/kg body weight/day for 13 weeks and reverse osmosis water was used as the control. All animals survived to the end of the study. During the experiment period, no abnormal changes were observed in clinical signs, body weight, or ophthalmological examinations. No adverse or test article-related differences were found in urinalysis, hematology, or serum biochemistry parameters between the treatment and control groups. Necropsy and histopathological examination indicated no treatment-related changes. According to the above results, the no-observed-adverse-effect level (NOAEL) of *L. rhinocerotis* was identified to be greater than 3400 mg/kg body weight (BW)/day in Sprague–Dawley rats.

## 1. Introduction

Mushroom fruiting bodies and mycelia have been recognized not only as a part of a regular diet with high nutrient value but also as a general health tonic for various ailments from time immemorial [1]. Extensive research on mushrooms has markedly increased, mainly due to their natural products with various biological activities [2]. They are reputed to exhibit medicinal benefits such as cholesterol-reducing, liver protective, immune-modulating, anti-diabetic, anti-inflammatory, anti-oxidant, anti-tumor, anti-microbial, and anti-viral activities [3]. With these various human health benefits, it is also fundamental to emphasize their toxicity characteristics as well as their safety for use in therapy or in human nutrition. 

*Lignosus rhinocerotis* (Cooke) Ryvarden, also known as Tiger’s Milk mushroom, is one of the most highly valued medicinal mushrooms sought after by the natives of Malaysia [4]. Its underground sclerotium (a compact of hardened fungal mycelium) is the part with medicinal value and has been used traditionally for the treatment of liver cancer, chronic hepatitis, and gastric ulcers [5]. Furthermore, recent research has demonstrated several bioactive properties of the sclerotium of *L. rhinocerotis* such as the enhancement of immune-modulatory actions on tumor cells [6], stimulatory effects on neurite outgrowth [7], anti-proliferative activities against carcinoma cells [8], and anti-acute inflammatory activities in the carrageenan-induced paw edema test [9]. Hence, there is a possibility of commercializing *L. rhinocerotis* sclerotium as a potential functional ingredient in food or dietary supplements.

Unfortunately, the supply of *L. rhinocerotis* sclerotium is limited as its presence can only be noticed when the fruiting body sprouts out from the ground [10]. Therefore, attempts have been made to cultivate this highly prized sclerotium or mycelium on agroresidues [11] or by liquid fermentation [12], respectively. However, the production of sclerotium usually takes several months [13], and it is often difficult to control the quality of the final product. For this reason, the potential of mycelium as a substitute for sclerotium concerning its bioactivities and chemical substitutes has received a great deal of attention. Furthermore, a recent study showed that *L. rhinocerotis* from liquid fermentation merits consideration as a potential substitute for the sclerotium from comparative studies on bioactivity evaluation and chemical profiling [12].

To date, results show that the *L. rhinocerotis* mycelium is devoid of its genotoxic effect under the experimental conditions [14] and its no-observed-adverse-effect level (NOAEL) is greater than 3400 mg/kg BW in reproductive and developmental animal studies using rats [15]. As part of an overall program to evaluate the effect and safety of this promising natural remedy for general use in food or health supplements, it is therefore necessary to carry out in-depth nutrition and safety evaluations. Thus, the present study was conducted to assess the nutritional and amino acid components as well as the 13-week subchronic toxicity in Sprague–Dawley rats, for demonstrating either long-term safety or prediction of acceptable daily intake (ADI). This study was performed in compliance with the testing guidelines of the Organization for Economic Cooperation and Development (OECD) and the US Food and Drug Administration (FDA) Good Laboratory Practice Regulations.

## 2. Materials and Methods

### 2.1. L. rhinocerotis Preparation

*Lignosus rhinocerotis* fruiting body was purchased from Ligno Biotech at Selangor, Malaysia (voucher code #0032). Mycelium was isolated and authenticated by sequencing using internal transcribed spacers (ITSs) 1 and 2. After our isolation demonstrated 99% similarity compared to the biological database using the Basic Local Alignment Search Tool (BLAST) (blast.ncbi.nlm.nih.gov/) (data not shown), the specimen was deposited at Bioresource Collection and Research Centre (BCRC) as BCRC 930202 in Taiwan. 

Fresh mycelium was transferred to fresh potato dextrose agar (PDA) plates every 15 days. A size of 1 cm^3^ was cut and inoculated into a 2-L Hinton flask containing 500 mL of nutrient medium consisting of 2% glucose, 0.5% soya bean powder, 0.2% peptone, and 0.1% yeast extract, with pH adjusted to 5.0 with 0.1 M hydrochloric acid. The flask was cultured at 100 rpm and incubated at 25 °C for 5 days before being transferred to a 500-L pilot seed fermenter containing 350 L of the same medium. After 4 days, this seed was transferred to a 50-ton full-scale fermenter and cultured under the same conditions. Mature mycelia were separated from the culture broth, lyophilized, ground to a powder, and stored at −20 °C. 

### 2.2. Proximate Analysis, Amino Acid Composition, and Umami Index

The proximate composition analysis, including crude protein, total crude lipid, ash, fiber, and moisture contents, of the freeze-dried mycelia was determined according to The Association of Official Analytical Chemists (AOAC) official methods 984.13, 43.275, 968.08, 991.43, and 950.46. B, respectively [16]. The energy values of the samples were determined by multiplying the protein content by 4, carbohydrate content by 4, and fat content by 9. The analysis of amino acid content was analyzed based on AOAC 994.12. The mushroom taste, also called the umami or palatable index, was determined using the ratio of aspartic and glutamic acids over the total amino acids according to a previous method [17].

### 2.3. Animals 

Forty-eight male and 48 female, six-week-old, Sprague-Dawley rats (BioLASCO, Taipei, Taiwan) were acclimated for 8 days before being randomly assigned to the control and three treatment groups of 12 rats per sex in each group. The body weight (BW) of the rats ranged from 196.2 to 236.9 g and from 145.7 to 173.4 g for the males and females, respectively. All animals were identified by an ear notch and were housed in pairs maintained under a relative humidity (55 ± 20%), controlled temperature (21 ± 2 °C), with alternating light cycles (12 h of light/dark). The frequency of ventilation was 10–15 times/h. During the study period, the animals had free ad libitum access to standard rodent diet (Laboratory Autoclavable Rodent Diet^®^ # 5010, PMI Nutrition International, Brentwood, MO, USA) and autoclaved, reverse-osmosis water. Aspen Chip bedding (Northeastern Products, Bellmore, NY, USA) was changed weekly. BW of all rats were measured before administration of the test article. All animal procedures were reviewed and approved by the Institutional Animal Care and Use Committee (IACUC, New Taipei City, Taiwan) at Level Laboratory Animal Center in Taiwan (approval No.: 120201-04).

### 2.4. Study Design

This study was performed based on the Organization for Economic Co-operation and Development (OECD) Guideline 408 [18] and was in strict accordance with the Good Laboratory Practice for Non-Clinical Laboratory Studies (FDA, 21 CFR, Part 58), Good Laboratory Practice for Non-Clinical Laboratory Studies (DOH, R.O.C., 3rd ed., 2006) and OECD Principles of Good Laboratory Practice (TAF OECD GLP Compliance, No. 1, 1997). The BWs of all rats were measured weekly until a scheduled necropsy 90 days later. Feed and water consumption were recorded weekly during the study period. *Lignosus rhinocerotis* mycelia (LRM) powder was given to rats daily by oral gavage at different dosages: 0 mg/kg BW/day (control), 850 mg/kg BW/day (low), 1700 mg/kg BW/day (medium), and 3400 mg/kg BW/day (high). LRM was prepared fresh daily and administered to each rat via a stainless-steel ball-tipped gavage needle. Clinical observations of mortality, morbidity, and possible signs of toxicity were made daily during the experimental period. At the end of the experiment, all surviving animals were anesthetized with a mixed solution of ketamine (80 mg/mL) and xylazine (8 mg/mL), followed by blood collection, exsanguination, and a necropsy. Gross necropsies included an examination of the external surface of the body, all thoracic and abdominal cavities, intestines, and visceral organs. 

### 2.5. Urinalysis

Before the gross necropsy examination, urine samples were collected approximately 12–16 h prior to terminal sacrifice using metabolism cages. Immediately after each urine sample was obtained, the pH, specific gravity, protein, glucose, ketone, bilirubin, urobilinogen, occult blood, leukocytes, and nitrite were analyzed with a urine analyzer (Bayer Clinitek-Status, Siemens, Malvern, PA, USA). Urine sediments were observed for red blood cells (RBCs), white blood cells (WBCs), cells, casts, crystals, and microbes using a Nikon E200 microscope (Tokyo, Japan).

### 2.6. Hematology and Serum Biochemistry 

Hematology and serum chemistry were performed after the 13-week dosing period. On the necropsy day, blood samples were obtained through the abdominal aorta and collected into three tubes that contained: (1) K2 EDTA for complete blood count analysis; (2) sodium citrate for a coagulation factor analysis; and (3) no anticoagulant for serum chemical analysis. Hematology examinations were performed using an automatic blood analyzer (Sysmex Xt-1800i, Sysmex, Kobe, Japan), which included analysis of the red blood cells (RBCs), white blood cells (WBCs), platelets, hemoglobin, hematocrit, mean corpuscular volume (MCV), mean corpuscular hemoglobin (MCH), mean corpuscular hemoglobin concentration (MCHC), neutrophils, eosinophils, basophils, monocytes, and lymphocytes. Anticoagulated blood samples were analyzed by blood coagulation analyzer (Sysmex CA-540, Siemens, Malvern, PA, USA) for the prothrombin time and activated partial thromboplastin time (APTT). An automated assay was performed with a model 7080 Hitachi analyzer (Tokyo, Japan) to test the following clinical chemical parameters: amylase, albumin, alkaline phosphatase (ALP), total bilirubin, alanine aminotransferase (ALT), aspartate aminotransferase (AST), gamma-glutamyl transferase (GGT), total protein, creatinine, blood urea nitrogen (BUN), cholesterol, triglycerides, creatine kinase, chloride, sodium, potassium, glucose, calcium, and phosphorus. 

### 2.7. Histopathology

The gross necropsy included examination of the external surface of the body, all thoracic and abdominal cavities, intestines, and visceral organs. The following tissues/organs were collected and weighed wet in situ after dissection: adrenals (paired), brain, epididymides (paired), heart, kidneys (paired), liver, ovaries with oviducts (paired), pituitary, spleen, testes (paired), thymus, and uterus with the cervix. The prostate and seminal vesicles with coagulating glands as a whole were weighed after fixation. Following the gross necropsy, these tissues/organs were fixed and preserved in 10% neutral buffered formalin or other appropriate fixatives for subsequent histopathological examination. Selected tissues in the control group and high dose group were trimmed, embedded, sectioned, and H&E-stained and then underwent a microscopic (Nikon E200, Tokyo, Japan) examination, according to a previous study [19].

### 2.8. Statistical Analysis 

Data collected from treated and control groups were compared by the one-way analysis of variance (ANOVA) method, followed by Dunnett’s method (SPSS, vers. 12.0, Illinois, Chicago, USA). A probability of 0.05 (*p* < 0.05) was used as the criterion of significance.

## 3. Results 

### 3.1. Proximate and Amino Acid Analysis of LRM Powder

The result of proximate analysis of freeze-dried LRM powder is shown in Table 1. The LRM powder contained 9.0 ± 4.2% moisture, 1.9 ± 1.3% ash, 1.6 ± 2.2% crude lipid, 8.4 ± 5.3% crude protein, 79.3 ± 4.6% carbohydrate, and 364 kcal/100 g energy. The total free amino acid ranged from 349 to 5636 mg/100 g and the umami index of freeze-dried LRM powder was 0.37 (Table 2).

### 3.2. Effect of LRM Powder on Body Weight and Feed Intake

**Figure 1:**
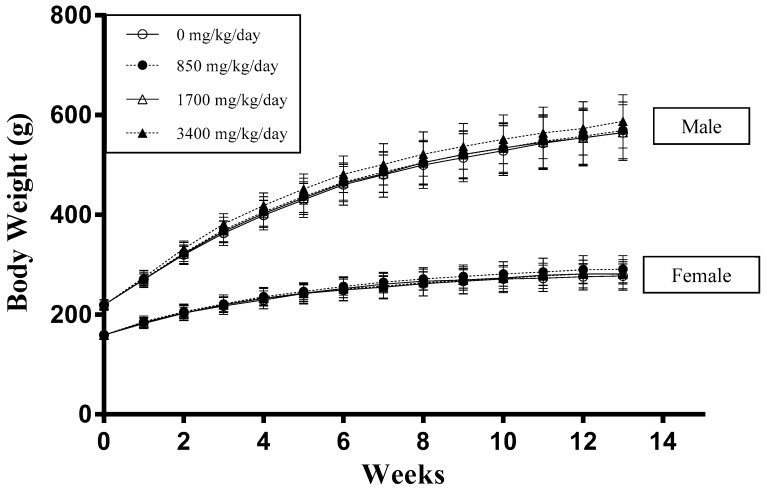
Effect of LRM powder on body weight of male and female Sprague-Dawley rats. Data expressed as mean ± SD (*n* = 12).

**Figure 2:**
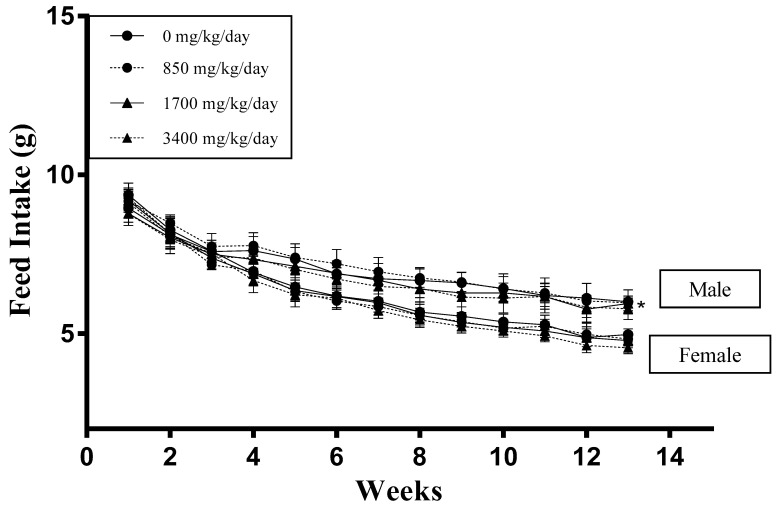
Effect of LRM powder on feed intake of male and female Sprague-Dawley rats. Data expressed as mean ± SD (*n* = 12).

Oral administration of LRM powder via gavage for 13 consecutive weeks showed no mortality or toxicity symptoms in any rats. Regarding body weight, there were no significant differences in any treated groups (Figure 1). There were also no significant differences among groups regarding feed intake except for male rats administered 3400 mg/kg/day LRM (*p* < 0.05) (Figure 2). This reduction was slight, reduced only in males, and was therefore considered to be an accidental change. 

### 3.3. Effect of LRM Powder in Hematological Parameters and Clinical Biochemistry

The summary of hematology results is presented in Table 3. No statistically significant changes were detected in the control and treated rats at all the doses tested. In addition, all clinical biochemistry remained unaltered in rats fed with LRM powder when compared to that of the untreated male and female rats (Table 4).

### 3.4. Effect of LRM Powder in Urinalysis

All urinary parameters in all groups were comparable among all the groups at the scheduled analysis, and there were no statistically or biologically significant changes between the treatment groups and the control group (Table 5).

### 3.5. Effect of LRM Powder in Necropsy, Organ Weight, and Histopathology 

Necropsy and macroscopic examination of organs and tissues from rats sub-chronically exposed to LRM showed no remarkable treatment-related damage. Moreover, no significant differences in the absolute weights of the measured organs were observed between any LRM-treated group and the concurrent control group for both sexes except an increased ovary with oviducts weight of female rats noted in the 850 mg/kg of LRM treatment groups at the end of the treatment period (Table 6). However, such differences were spontaneous, non-dose-dependent, and therefore not considered toxicologically or biologically relevant.

The histopathological lesions of the highest dose and control groups included minimal focal round cell collection in stromal cells of the harderian glands, focal minimal cysts in the ultimobranchial of the thyroid, focal minimal-to-mild myocarditis in the heart, minimal focal round cell collection in stromal cells of the pancreas, minimal multifocal clear cells in the liver, minimal focal round cell collection in the cortex of the kidneys, minimal-to-mild focal round cell collection in the stromal cells of the prostate, and minimal focal round cell collection in the ovaries and oviduct (Table 7). However, considering the incidence and degree of their occurrence, they were considered to be the frequently spontaneous pathological changes in this strain of this age, and were not related to the administration of LRM.

## 4. Discussion

Recently, medicinal mushrooms have become a popular dietary supplement to promote health [21]. Considering dietary supplements are likely to be used for a long-term duration, it is necessary to perform appropriate preclinical toxicity assessments to predict toxicity and to select a safe dose for human health. According to previous studies, research on *L. rhinocerotis* bioactivities and toxicities have mainly focused on the naturally occurring sclerotium [22] and may have overlooked mushroom mycelia from submerged liquid fermentation, which ensures a high uniform quantitative biomass production as well as a high biological value [23]. Using principal component analysis, the chemical profiles revealed that the *L. rhinocerotis* mycelial extracts cultured under different conditions of liquid fermentation were distinct from those of the sclerotium [12]. As *L.* rhinocerotis from liquid fermentation merits consideration as a potential substitute for the sclerotium, toxicity testing is imperative to identify the types of adverse effects that may occur and to predict exposures in humans that should be without risk.

In our previous study, the genotoxicity [14] and prenatal developmental toxicity [15] were conducted on LRM and showed no significant findings that lead to meaningful interpretation of toxic effect. However, a subchronic toxicity evaluation is necessary as it can provide valuable information about the toxicity of LRM and can provide suitable dose regimens for long-term studies. Hence, for the first time, the present study investigated the potential toxicity of LRM in rats after 13 weeks of repeated oral administration at concentrations of 0, 850, 1700, and 3400 mg/kg/day. 

Given that LRM was developed as a health supplement, the amino acid and proximate analyses of LRM were first measured, as they are important indices to classify the nutritional value of a food material [24]. In amino acid analysis, the LRM contained a total of 21,728 mg/100 g, with the abundant amino acids being glutamate (5636 mg), aspartate (2384 mg), and arginine (1766 mg). Similar to previously reported *L. rhinocerotis* sclerotia, all essential amino acids, except for tryptophan, were present [25]. Moreover, earlier studies showed that major constituents of sclerotia were carbohydrates (82.60 ± 0.01%), protein (7.02 ± 0.20%), and fat (0.49 ± 0.00%) with an energy of 362.83 ± 0.76 kcal/kg [25]. In this study, the carbohydrate (79.3 ± 4.6%), protein (8.4 ± 5.3%), and fat content (1.6 ± 2.2%) of LRM were comparable to those of the reported sclerotia, providing a basis for considering the mycelium as an alternative to the sclerotium. Further, these values are in a similar range to those reported from a previous study (73.01 ± 0.04% carbohydrates, 7.87 ± 0.01% protein, 2.30 ± 0.06% fat, and 344.20 ± 0.71 energy) [25]. According to the results of a recent clinical trial, a high level of plant-based carbohydrates and low-fat diet for 16 weeks was found to reduce body weight and body fat as well as to improve insulin function in overweight individuals [26]. Considering that LRM has a high level of carbohydrates and very low level of lipids, these findings suggested that LRM could replace sclerotium and fits the nutritional requirement for those who are on a weight-management program.

As some mushrooms are bitter [27], consideration should also be given to the taste of medicinal mushrooms for supplement formulation. Among amino acids, aspartic and glutamic acid are classified as monosodium glutamate-like (MSG-like) components, which give mushroom a peculiar umami or palatable taste. In a previous study, the ratio of umami amino acids to total amino acids (umami index) of various mushrooms ranged between 0.21 and 0.32 [28]. A higher umami index (0.37), however, was found in this study. A possibility is that *L. rhinocerotis* contributes protease and peptidase during fermentation, which increases the formation of these taste-active amino acids [29]. Umami substances via G protein-coupled receptors and heteromeric T1R1 + T1R3 receptors not only could elicit umami taste but also exert many health benefits, including fat regulation [30]. Considering that LRM provides not only rich nutrients, but also gives a sour and umami taste, which contributes a pleasant taste to the food, it thus could be used as a natural source for development and application in food products. 

When no human data on LRM are available, animal data are essential for a safety assessment. In this study, treatment with LRM for 13 weeks at up to 3400 mg/kg/day was not associated with adverse effects on toxicologically-relevant parameters in rats of either sex. Although there was a linearly decreased feed intake in both the control and the treatment groups during the study period, no significant differences between the groups were found as compared to the control group, except for the male rats in the high-dose group. These decreases may be explained by the fact that animals regulate their intake according to dietary energy level [31]. Studies have shown that consumption of a high-energy diet could decrease the level of adenosine monophosphate-activated protein kinase (AMPK) expression in the hypothalamus, resulting in reduced hunger and enhanced satiety [32,33]. In line with these studies, as the commercial diet and LRM both contained high energy (with 3.42 and 3.34 kcal/g, respectively), the results of this study suggest that rodents can adjust their feed intake according to the energy state of the diet and thereby decreased feed intake. 

Despite decreased feed intake, there was no change directly proportional to body weight. Alterations in body weight are sensitive indicators of adverse effects of drugs and chemicals [34]. After 13 weeks of treatment of LRM, all the animals exhibited a normal increment in body weight, suggesting that LRM did not interfere with the normal metabolism of animals. Moreover, there were no significant differences among the treatment groups based on the hematological and serum chemical parameters analyses, indicating that exposure to LRM caused no injury to the hematopoietic, hepatic, cardiac, and renal systems [35]. Further, in urinalysis, there were no treatment-related effects for both sexes in the treatment groups, demonstrating that LRM produces well-functioning excretory systems. 

Organ weight changes are indicators of possible morphological or functional changes. In this study, no significant changes in the weight of the brain, heart, kidneys, liver, spleen, or testes were observed. Although ovaries with oviducts weight (0.14233 ± 0.02466) in the low-dose group were slightly higher than those of the control group (0.11606 ± 0.01284) after 13 weeks, these changes lack dose-relationship and treatment-related histological findings. Hence, these differences were considered to be normal biological variation. 

In the histopathological examination, the lesions found in the 3400 mg/kg LRM groups include focal round cell collection (harderian glands, pancreas, kidney, prostate, and ovaries and oviduct), cysts (thyroid), inflammation (lung and heart), degeneration (liver and kidney), and tubular lumen mineralization (kidney), which are common in repeated toxicity studies [20]. However, these lesions were also observed in the control group with similar frequency and severity grade. Considering that these lesions were minor, did not follow a dose-dependent pattern, and within normal ranges in all the animals, it can be concluded that changes might have been background lesions and not a treatment-related effect.

## 5. Conclusions

Based on the findings of this study, dietary administration of LRM to Sprague-Dawley rats over a period of 13 weeks resulted in a no-observed-adverse-effect level (NOAEL) of 3400 mg/kg BW/day. To extrapolate the results from rats to humans, a default uncertainty factor of 100 was applied and the ADI for LRM can be estimated as 34 mg/kg BW/day. Although the findings may be used in the future for reference, initial clinical trials involving healthy adult volunteers are required to assure both the safety and efficacy of LRM. 

## Figures and Tables

**Table 1:** Proximate composition of *L. rhinocerotis* mycelium (LRM) powder.

	Proximate Analysis (%)
Moisture	9.0 ± 4.2
Ash	1.9 ± 1.3
Crude lipid	1.6 ± 2.2
Crude protein	8.4 ± 5.3
Carbohydrate	79.3 ± 4.6
Energy (kcal/100 g)	364.0 ± 22.6

Each value is expressed in mean ± SD (*n* = 2.).

**Table 2:** The amino acid composition and the umami index of freeze-dried LRM powder.

Amino Acid	mg/100 g
Aspartate	2384
Glutamate	5636
Serine	1007
Histidine	588
Glycine	1106
Threonine	1054
Alanine	1218
Arginine	1766
Tyrosine	436
Cystine	349
Valine	753
Methionine	360
Phenylalanine	718
Isoleucine	764
Leucine	1205
Lysine	1355
Proline	1030
Umami index ^a^	0.37

^a^ Ratio of aspartic and glutamic acids to total amino acids, calculated according to a previous method [17].

**Table 3:** Effects of LRM powder on hematology in male and female Sprague-Dawley rats after 13 weeks.

Parameters	Control (Distilled Water)	*Lignosus rhinocerotis* Mycelium (mg/kg)		
		850	1700	3400
**Male**				
WBC (10^3^/μL)	5.648 ± 1.007	6.916 ± 2.754	7.327 ± 1.820	6.308 ± 1.258
RBC (10^6^/μL)	9.288 ± 0.383	9.343 ± 0.412	9.464 ± 0.360	9.173 ± 0.363
Hemoglobin (g/dL)	16.00 ± 0.44	16.26 ± 0.54	16.36 ± 0.63	15.93 ± 0.51
Hematocrit (%)	44.69 ± 1.16	45.41 ± 1.32	45.47 ± 1.63	44.67 ± 1.68
MCV (fL)	48.18 ± 1.87	48.64 ± 1.56	48.08 ± 1.89	48.75 ± 2.09
MCH (pg)	17.25 ± 0.63	17.44 ± 0.49	17.29 ± 0.55	17.38 ± 0.58
MCHC (g/dL)	35.79 ± 0.48	35.80 ± 0.29	35.98 ± 0.64	35.68 ± 0.66
Platelet (10^3^/μL)	1136.8 ± 109.6	1169.5 ± 79.9	1174.2 ± 151.6	1189.7 ± 154.5
Neutrophil (%)	29.41 ± 8.18	31.87 ± 5.91	34.77 ± 9.05	30.28 ± 10.78
Lymphocyte (%)	65.73 ± 9.12	63.24 ± 6.09	60.37 ± 9.33	64.21 ± 11.16
Monocyte (%)	4.59 ± 1.61	4.62 ± 0.89	4.66 ± 0.82	5.18 ± 1.45
Eosinophil (%)	0.28 ± 0.28	0.26 ± 0.15	0.20 ± 0.13	0.34 ± 0.30
Basophil (%)	0.00 ± 0.00	0.02 ± 0.04	0.01 ± 0.03	0.00 ± 0.00
PT (sec)	11.17 ± 1.34	12.02 ± 2.28	11.88 ± 1.83	12.54 ± 2.02
APTT (sec)	16.88 ± 1.40	17.28 ± 1.78	17.36 ± 1.07	17.28 ± 1.19
**Female**				
WBC (10^3^/μL)	4.893 ± 1.842	5.020 ± 2.372	6.608 ± 2.365	6.259 ± 3.018
RBC (10^6^/μL)	8.277 ± 0.482	8.466 ± 0.403	8.501 ± 0.355	8.501 ± 0.294
Hemoglobin (g/dL)	15.20 ± 0.29	15.45 ± 0.58	15.61 ± 0.62	15.50 ± 0.59
Hematocrit (%)	42.71 ± 0.41	43.49 ± 1.52	43.94 ± 1.74	43.46 ± 1.35
MCV (fL)	51.75 ± 2.82	51.40 ± 1.28	51.73 ± 1.88	51.15 ± 1.49
MCH (pg)	18.40 ± 0.74	18.25 ± 0.35	18.37 ± 0.51	18.23 ± 0.37
MCHC (g/dL)	35.58 ± 0.59	35.52 ± 0.41	35.53 ± 0.50	35.65 ± 0.62
Platelet (10^3^/μL)	1061.5 ± 89.1	1066.1 ± 118.4	1065.9 ± 134.7	1110.2 ± 85.1
Neutrophil (%)	16.51 ± 3.56	15.43 ± 4.20	16.76 ± 6.27	19.76 ± 10.38
Lymphocyte (%)	79.40 ± 4.06	80.83 ± 4.51	79.27 ± 6.50	76.02 ± 10.83
Monocyte (%)	3.83 ± 1.27	3.48 ± 1.09	3.73 ± 0.81	3.98 ± 0.86
Eosinophil (%)	0.27 ± 0.29	0.28 ± 0.22	0.23 ± 0.14	0.23 ± 0.28
Basophil (%)	0.00 ± 0.00	0.00 ± 0.00	0.02 ± 0.04	0.02 ± 0.06
Reticulocyte (%)	9.34 ± 0.24	9.36 ± 0.28	9.22 ± 0.26	9.36 ± 0.15
APTT (sec)	14.71 ± 0.95	14.45 ± 0.83	14.72 ± 0.81	15.13 ± 0.90

Data were expressed as mean ± SD (*n* = 12). White blood cell (WBC); Red blood cell (RBC); Mean corpuscular volume (MCV); Mean corpuscular hemoglobin (MCH); Mean corpuscular hemoglobin concentration (MCHC); Activated partial thromboplastin time (APTT).

**Table 4:** Effects of LRM powder on clinical biochemistry in male and female Sprague-Dawley rats after 13 weeks.

Parameters	Control (Distilled Water)	*Lignosus rhinocerotis* Mycelium (mg/kg)		
		850	1700	3400
**Male**				
AST (U/L)	117.96 ± 22.63	122.56 ± 17.88	127.61 ± 26.56	129.73 ± 16.56
ALT (U/L)	32.86 ± 5.69	30.06 ± 4.30	29.24 ± 4.41	31.93 ± 11.01
Glucose (mg/dL)	152.68 ± 19.85	167.53 ± 42.16	154.78 ± 22.24	160.41 ± 19.35
Total protein (g/dL)	6.13 ± 0.32	6.14 ± 0.36	6.09 ± 0.37	6.01 ± 0.40
Albumin (g/dL)	4.06 ± 0.22	3.98 ± 0.23	3.99 ± 0.34	3.94 ± 0.25
Total bilirubin (mg/dL)	<0.04	<0.04	<0.04	0.05
BUN (mg/dL)	15.36 ± 1.80	15.49 ± 2.09	14.16 ± 2.70	14.86 ± 2.09
Creatinine (mg/dL)	0.50 ± 0.04	0.51 ± 0.09	0.46 ± 0.07	0.54 ± 0.11
GGT (U/L)	<2.0	<2.0	<2.0	<2.0
Alkaline phosphatase (U/L)	256.92 ± 68.56	265.83 ± 57.40	0.63 ± 55.61	236.48 ± 46.79
Cholesterol (mg/dL)	61.32 ± 14.20	64.13 ± 14.50	49.78 ± 9.82	53.70 ± 11.30
Triglyceride (mg/dL)	20.43 ± 5.69	26.18 ± 15.45	23.39 ± 14.68	23.13 ± 7.58
Calcium (mg/dL)	9.49 ± 0.52	9.63 ± 0.79	9.53 ± 0.78	9.34 ± 0.57
Phosphorus (mg/dL)	6.74 ± 1.06	7.11 ± 1.10	6.73 ± 0.92	6.43 ± 0.92
Creatine kinase (U/L)	428.93 ± 173.14	483.89 ± 165.51	382.94 ± 168.04	414.63 ± 182.19
Amylase (U/L)	1306.3 ± 180.20	1325.80 ± 240.70	1391.80 ± 196.20	1329.30 ± 164.10
Sodium (mmol/L)	145.68 ± 6.03	143.60 ± 5.92	144.63 ± 9.87	144.28 ± 5.00
Potassium (mmol/L)	4.38 ± 0.20	4.41 ± 0.25	4.40 ± 0.35	4.58 ± 0.27
Chloride (mmol/L)	101.29 ± 4.99	99.66 ± 5.27	100.95 ± 8.08	100.53 ± 3.81
**Female**				
AST (U/L)	80.74 ± 9.98	97.22 ± 28.79	84.07 ± 17.49	85.07 ± 23.27
ALT (U/L)	19.91 ± 5.28	23.31 ± 6.43	19.11 ± 3.41	34.12 ± 53.87
Glucose (mg/dL)	126.01 ± 24.32	137.93 ± 31.11	124.50 ± 27.44	124.53 ± 24.10
Total protein (g/dL)	5.88 ± 0.68	6.38 ± 0.89	5.91 ± 0.95	5.99 ± 0.83
Albumin (g/dL)	4.14 ± 0.47	4.48 ± 0.63	4.12 ± 0.52	4.22 ± 0.54
Total bilirubin (mg/dL)	0.07 ± 0.02	0.07 ± 0.03	0.08 ± 0.03	0.06 ± 0.01
BUN (mg/dL)	14.62 ± 3.12	15.13 ± 3.15	14.30 ± 1.78	12.69 ± 2.40
Creatinine (mg/dL)	0.52 ± 0.07	0.57 ± 0.16	0.50 ± 0.06	0.47 ± 0.10
GGT (U/L)	<2.0	<2.0	<2.0	<2.0
Alkaline phosphatase (U/L)	118.96 ± 43.98	128.16 ± 43.78	110.61 ± 38.00	122.11 ± 28.83
Cholesterol (mg/dL)	53.69 ± 16.20	60.08 ± 19.43	59.78 ± 11.48	54.46 ± 18.70
Triglyceride (mg/dL)	17.86 ± 8.31	23.94 ± 15.83	14.71 ± 4.72	19.44 ± 14.77
Calcium (mg/dL)	9.03 ± 0.82	9.68 ± 1.41	8.87 ± 1.26	8.90 ± 1.08
Phosphorus (mg/dL)	5.78 ± 1.02	5.85 ± 2.46	5.58 ± 0.90	5.20 ± 0.78
Creatine kinase (U/L)	301.18 ± 79.02	397.21 ± 179.35	338.78 ± 171.80	332.68 ± 196.74
Amylase (U/L)	849.70 ± 169.90	1028.80 ± 314.60	852.70 ± 240.50	815.10 ± 149.50
Sodium (mmol/L)	133.42 ± 13.66	142.69 ± 16.82	134.54 ± 14.85	134.18 ± 12.54
Potassium (mmol/L)	4.35 ± 0.90	4.80 ± 1.96	4.04 ± 0.57	4.09 ± 0.43
Chloride (mmol/L)	97.38 ± 11.93	104.09 ± 13.38	97.19 ± 11.12	97.61 ± 9.54

Data were expressed as mean ± SD (*n* = 12). Aspartate aminotransferase (AST); Alanine aminotransferase (ALT); Alkaline phosphatase (ALP); Gamma-glutamyl transferase (GGT); Blood urea nitrogen (BUN).

**Table 5:** Effects of *L. rhinocerotis* mycelium on urinalysis parameters of male and female Sprague-Dawley rats after 13 weeks.

Parameters	Control (Distilled Water)	*Lignosus rhinocerotis* Mycelium (mg/kg)		
		850	1700	3400
**Male**				
Volume (mL)	19.08 ± 8.16	17.46 ± 8.18	19.46 ± 10.57	16.79 ± 6.08
Specific Gravity	1.017 ± 0.004	1.015 ± 0.003	1.018 ± 0.004	1.019 ± 0.004
pH	7.29 ± 0.45	7.08 ± 0.19	7.13 ± 0.23	7.00 ± 0.30
Urobilinogen (EU/dL)	0.20 ± 0.00	0.20 ± 0.00	0.20 ± 0.00	0.20 ± 0.00
**Female**				
Volume (mL)	12.92 ± 8.27	12.63 ± 5.53	10.83 ± 4.74	14.46 ± 9.54
Specific Gravity	1.016 ± 0.004	1.017 ± 0.004	1.018 ± 0.003	1.016 ± 0.004
pH	7.00 ± 0.21	6.96 ± 0.14	6.92 ± 0.19	7.00 ± 0.37
Urobilinogen (EU/dL)	0.33 ± 0.31	0.20 ± 0.00	0.20 ± 0.00	0.27 ± 0.23

Data were expressed as mean ± SD (*n* = 12).

**Table 6:** Effects of *L. rhinocerotis* mycelium on organ weight of male and female Sprague-Dawley rats after 13 weeks.

Parameters	Control (Distilled Water)	*Lignosus rhinocerotis* Mycelium (mg/kg)		
		850	1700	3400
**Male**				
Adrenals (Paired)	0.053 ± 0.005	0.058 ± 0.008	0.054 ± 0.009	0.057 ± 0.007
Brain	2.177 ± 0.091	2.158 ± 0.069	2.183 ± 0.058	2.113 ± 0.087
Pituitary	0.014 ± 0.002	0.013 ± 0.001	0.013 ± 0.001	0.013 ± 0.001
Thymus	0.421 ± 0.122	0.389 ± 0.081	0.421 ± 0.099	0.383 ± 0.108
Heart	1.583 ± 0.152	1.575 ± 0.138	1.538 ± 0.110	1.705 ± 0.149
Kidney (Paired)	3.468 ± 0.389	3.468 ± 0.364	3.588 ± 0.341	3.519 ± 0.458
Liver	14.519 ± 1.988	14.548 ± 2.409	14.750 ± 1.633	15.286 ± 2.676
Spleen	0.860 ± 0.195	0.918 ± 0.213	0.815 ± 0.174	0.854 ± 0.165
Testes (Paired)	3.493 ± 0.258	3.370 ± 0.355	3.281 ± 0.140	3.214 ± 0.376
Epididymides (Paired)	1.448 ± 0.172	1.374 ± 0.126	1.434 ± 0.060	1.393 ± 0.129
Prostates and seminal vesicles with coagulating glands	3.803 ± 0.427	3.600 ± 0.321	3.580 ± 0.449	3.630 ± 0.394
**Female**				
Adrenals (Paired)	0.069 ± 0.016	0.068 ± 0.013	0.065 ± 0.011	0.070 ± 0.009
Brain	1.939 ± 0.064	1.995 ± 0.079	1.911 ± 0.108	1.945 ± 0.080
Pituitary	0.016 ± 0.003	0.015 ± 0.002	0.015 ± 0.003	0.015 ± 0.003
Thymus	0.256 ± 0.061	0.291 ± 0.050	0.285 ± 0.070	0.301 ± 0.075
Heart	0.898 ± 0.089	0.909 ± 0.053	0.874 ± 0.092	0.912 ± 0.060
Kidney (Paired)	1.845 ± 0.337	1.881 ± 0.163	1.846 ± 0.192	1.903 ± 0.192
Liver	7.303 ± 0.762	7.703 ± 0.658	7.193 ± 0.777	7.486 ± 0.536
Spleen	0.487 ± 0.077	0.507 ± 0.075	0.529 ± 0.106	0.524 ± 0.104
Ovaries with oviducts	0.116 ± 0.013	0.142 ± 0.0247 *	0.135 ± 0.027	0.136 ± 0.018
Uterus with cervix	0.817 ± 0.318	0.676 ± 0.227	0.639 ± 0.163	0.696 ± 0.249

Data were expressed as mean ± SD (*n* = 12); * Statistically significant (*p* < 0.05) when compared to the control group.

**Table 7:** Histological findings.

Parameters		Control (Distilled Water)		*Lignosus rhinocerotis* Mycelium (High Dose)	
Gender		Male	Female	Male	Female
Histopathologic Findings	Severity ^a^	(N/N) ^b^			
**Harderian glands**					
Focal round cell collection	1	0/12	2/12	1/12	0/12
**Thyroid**					
Cyst, ultimobranchial, focal	1	1/12	2/12	2/12	1/12
**Lung**					
Alveolar histiocytosis, focal	1	2/12	2/12	1/12	2/12
**Heart (Aorta)**					
Myocarditis, focal	1–2	2/12	0/12	1/12	0/12
**Pancreas**					
Focal round cell collection	1	1/12	0/12	1/12	0/12
**Liver**					
Clear cell, multifocal	1	0/12	0/12	2/12	0/12
Degeneration/Necrosis, focal.	1	0/12	0/12	1/12	0/12
**Kidneys**					
Focal round cell collection, cortex	1	2/12	0/12	0/12	1/12
Cystic degeneration, medulla, focal	1	1/12	0/12	0/12	0/12
Mineralization, tubular lumen, focal	1	0/12	1/12	0/12	2/12
**Prostate**					
Focal round cell collection	1–2	5/12	-	5/12	-
**Ovaries and oviduct**					
Focal round cell collection	1	-	2/12	-	2/12

^a^ Severity grading according to a previous study [20]. 1 = minimal (<10%), 2 = mild (10–39%), 3 = moderate (40–79%), 4 = marked (80–100%); ^b^ Animal numbers with histopathologic findings/Animal numbers examined.
